# Cognitive Health Worries, Reduced Physical Activity and Fewer Social Interactions Negatively Impact Psychological Wellbeing in Older Adults During the COVID-19 Pandemic

**DOI:** 10.3389/fpsyg.2022.823089

**Published:** 2022-02-17

**Authors:** Emma Sutton, Jonathan Catling, Katrien Segaert, Jet Veldhuijzen van Zanten

**Affiliations:** ^1^School of Psychology, College of Life and Environmental Sciences, University of Birmingham, Birmingham, United Kingdom; ^2^Centre for Developmental Science, School of Psychology, College of Life and Environmental Sciences, University of Birmingham, Birmingham, United Kingdom; ^3^Centre for Human Brain Health, School of Psychology, College of Life and Environmental Sciences, University of Birmingham, Birmingham, United Kingdom; ^4^School of Sport, Exercise and Rehabilitation Sciences, College of Life and Environmental Sciences, University of Birmingham, Birmingham, United Kingdom

**Keywords:** cognitive health worries, COVID-19, older adults, psychological wellbeing, physical activity, social interaction

## Abstract

The Coronavirus pandemic has significantly affected psychological wellbeing in older adults, with cases of depression, anxiety and loneliness rising in the general population. Cognitive health has also potentially been affected, as social isolation can lead to cognitive decline. Worrying about cognitive health can be damaging to psychological wellbeing and is especially relevant to explore in the context of the Coronavirus pandemic. The objective of the present study was to explore the associations between cognitive health worries and wellbeing, and to investigate whether physical activity and social contact can mitigate negative effects of the pandemic on psychological wellbeing. Older adults (*N* = 191) completed an online survey which included measures of cognitive health worries, depression, anxiety, loneliness, social isolation, fatigue, impact of the Coronavirus pandemic, quality of life, subjective vitality, and physical activity. Analyses indicated that cognitive health worries, lower levels of physical activity and smaller amounts of social interaction were associated with poorer psychological and physical wellbeing. Results showed that worrying about cognitive health is associated with poorer wellbeing, and so interventions are needed to encourage positive cognitive functioning in times of social isolation. Promoting physical activity and social interaction is also beneficial, as results show that exercise and social contact are linked with improved wellbeing.

## Introduction

The ongoing Coronavirus (COVID-19) pandemic has had a profound effect on peoples’ lives across the world. Older adults in particular are more at risk of serious illness, further complications, and death as a result of COVID-19; over 65s account for more than 9 out of 10 COVID-19 related deaths in the United Kingdom (ONS, 2020a). As such, older adults were some of the first people to be advised to stay at home, “shielding” themselves from the disease.

While this social isolation may have protected them from COVID-19, it directly disrupted activities that many older adults take part in, such as seeing family or friends, volunteering and everyday physical exercise routines. Shielding and social distancing has undoubtedly protected older adults from catching the virus, but the resulting social isolation has potentially harmed their physical and psychological wellbeing (Smith et al., 2020). Furthermore, extended periods of isolation are known to harm cognitive health (Lara et al., 2019) and worrying about cognitive decline can be common in older populations (Bowen et al., 2019).

Research completed during the pandemic shows a significant impact on psychological wellbeing; rates of depression and anxiety are reported to be between 16–28% of the general population (Rajkumar, 2020). The percentage of United Kingdom adults reporting moderate or severe symptoms of depression has almost doubled from July 2019 (9.7%) to June 2020 (19.2%), with 12.9% of adults reporting that these symptoms developed during the pandemic (ONS, 2020b). Social isolation appears to be a contributing factor to poor mental health, with the prevalence of loneliness now at 27% of the population (Groarke et al., 2020). Those who are especially socially isolated seem to be the worst affected; one study found that older adults who live alone were twice as likely to report symptoms of loneliness, and that when shielding (staying at home and minimising face-to-face contact with others), 50% reported a decline in quality-of-life, and 40% a decline in mobility (Bailey et al., 2021). Higher loneliness scores have been found to correlate positively with symptoms of depression and anxiety (Robb et al., 2020), and another study found that increased loneliness scores predicted scores of depression in older adults (Krendl and Perry, 2021).

Recent research has looked at possible protective factors that might limit these negative effects. Physical activity is associated with better psychological wellbeing (Deslandes et al., 2009). This has continued during the pandemic: older adults who managed to meet the recommended guidelines for physical activity during the pandemic reported fewer depressive symptoms and higher positive affect compared to those who did not meet guidelines (Carriedo et al., 2020). Social contact is known to be another protective factor in both mental (Schwartz and Litwin, 2019) and cognitive (Crooks et al., 2008) health. Co-habiting and living with other people seem to help psychological wellbeing, particularly loneliness (Groarke et al., 2020). It is evident that staying physically active helps to mitigate some of the negative mental health effects of social isolation during the pandemic. Similarly, regular contact with loved ones appears to be a buffer against declines in psychological wellbeing. In this sense, older adults can still feel connected socially, despite following social distancing measures (Damiot et al., 2020). The present study aimed to investigate the benefits of physical activity and social contact on psychological wellbeing, in a sample of older adults during the COVID-19 pandemic.

Being concerned about the pandemic, termed “COVID-related anxiety” (Shevlin et al., 2020) may also have an impact on psychological wellbeing and social isolation. Compared to younger participants, older adults report higher levels of anxiety about COVID-19 (Hyland et al., 2020; Shevlin et al., 2020), and higher levels of COVID-related anxiety have been shown to be predictive of poorer psychological wellbeing in a sample of 18–49-year-olds (Aslam et al., 2021). This poorer mental wellbeing can be counteracted by physical activity, emphasising the benefits of keeping physically active in the pandemic (Wright et al., 2021). However, to our knowledge, there is no research looking at the effects of COVID-related anxiety on psychological wellbeing in adults over 60.

We know that in older adults there is a natural decline in cognitive functioning (e.g., Salthouse, 2010). However, this decline can be moderated by lifestyle factors such as physical activity (Cheng, 2016) and social interaction (Kuiper et al., 2015; Bzdok and Dunbar, 2020). Accurate measures of cognitive ability are more difficult to obtain in online or remote psychological studies. However, remote versions of validated cognitive tools, such as the telephone version of the Mini-Mental State Examination, have been used for some time and have been utilised specifically during the pandemic to the same success as in-person versions (e.g., Quattropani et al., 2021).

Other studies conducted during the pandemic have explored self-reported memory complaints as a way of reporting cognitive functioning. Only a small percentage of older participants (8%) report that their cognitive functioning had declined as a result of COVID-19 (De Pue et al., 2021), with another study finding that while young participants’ subjective memory complaints have increased compared to pre-pandemic, older adults were low and remained stable (Fiorenzato et al., 2021). A mediating factor may be the amount of social isolation participants report. A recent longitudinal study found that older adults who were isolated both before and during the pandemic, and older adults who became isolated as a result of the pandemic, were significantly more likely to self-report cognitive decline compared to those that remained non-isolated (Noguchi et al., 2021). These subjective declines in cognitive functioning due to the pandemic appear to have a significant relationship with depression (De Pue et al., 2021; Fiorenzato et al., 2021) and anxiety (Fiorenzato et al., 2021) symptoms, indicating that those who are worried about their cognitive abilities may be concerned to the point of it impacting their psychological wellbeing.

An alternative way to think about cognitive health may be to look at self-reported worries about brain health. These cognitive health worries have mainly been used in the context of developing dementia, otherwise known as “dementia worry.” Dementia worry can be widespread in the general population, ranging from 26–60% of reported populations (Kessler et al., 2012; Bowen et al., 2019), and can be exacerbated further by genetic exposure to dementia (Lee et al., 2020). Interestingly, research shows a reverse-U shaped relationship with age, with dementia worry peaking at 70 years old (Bowen et al., 2019).

Cognitive health worries have been associated with poorer psychological wellbeing. A study exploring worries about cognitive functioning and concerns about developing dementia found that participants who were more concerned about their cognitive health had higher levels of depression and stress, and poorer life satisfaction scores (Cutler and Bragaru, 2017). Participants who worry about developing dementia tend to report poorer cognitive functioning (Cutler and Hodgson, 1996), and high scores of dementia worry have been linked to poorer cognitive functioning, shown by significantly lower scores of executive functioning (Caughie et al., 2021). While asking participants if they are worried about their cognitive health does not objectively measure cognitive ability, it does relate to cognitive performance and can negatively impact psychological wellbeing (Cutler and Bragaru, 2017; Caughie et al., 2021).

These cognitive health worries have yet to be explored in the context of the pandemic. As mentioned above, extended periods of social isolation can lead to cognitive decline (Lara et al., 2019; Noguchi et al., 2021; Yu et al., 2021) and so it may be reasonable to theorise that these cognitive worries have increased since the start of the pandemic. Older adults are hesitant to return to their normal day-to-day lives (Age UK, 2020) and this extended isolation could lead to further declines in cognitive and psychological wellbeing.

The first aim of the current study was to examine whether cognitive health worries were associated with poorer psychological and physical wellbeing in older adults during the COVID-19 pandemic. A second aim was to corroborate previous findings conducted during the pandemic, specifically that greater levels of physical activity and social contact are associated with better mental health. A final aim was to expand research into COVID-related anxiety and its impact on wellbeing.

We hypothesised that first, worrying about one’s cognitive health would have a significant negative association with psychological and physical wellbeing. Second, we hypothesised that higher levels of physical activity and social contact would be significantly correlated with better psychological wellbeing. Finally, we hypothesised that concern about COVID-19 would have a negative association with psychological wellbeing.

## Materials and Methods

### Participants

A total of 191 participants (75 male, 114 female, 2 prefer not to answer), recruited through social media, local community groups, and word of mouth, completed the entire questionnaire. A further 51 participants started the questionnaire but did not complete all questions and were therefore not included in the present analyses. All participants were over 60 years of age and lived in the United Kingdom, with a mean age of 70.5 years (SD 6.4, age range 60–88). The majority (*N* = 183) identified as White, married (*N* = 141) and described their living arrangements as living with a partner or spouse (*N* = 126). Full demographics are shown in Table 1.

**TABLE 1:** Participant demographics.

Demographic category	Frequency	Percentage
**Education level**		
Primary school	4	2.1
GCSEs, O levels or equivalent	41	21.5
A-Levels or equivalent	26	13.6
University undergraduate programme (e.g., BSc, BA)	54	28.3
University postgraduate programme (e.g., MSc, MA)	54	28.3
Doctoral degree (e.g., PhD)	4	2.1
Other	7	3.7
Prefer not to answer	1	0.5
**Ethnicity**		
White	183	95.8
Mixed/multiple ethnic groups	3	1.6
Asian Chinese/Asian Indian/Asian Pakistani or Bangladeshi/Asian other	1	0.5
European	1	0.5
Prefer not to answer	3	1.6
**Marital status**		
Married	141	73.8
Widowed	20	10.5
Divorced	15	7.9
Single (never married)	11	5.8
Legally separated	2	1.0
Prefer not to answer	2	1.0
**Living arrangements**		
Live with partner or spouse	126	66.0
Live with family members	28	14.7
Live alone	25	13.1
Live alone but part of a support bubble^a^	11	5.8
Prefer not to answer	1	0.5

*^a^A support bubble in this instance referred to the United Kingdom Government definition of the term, in which those who lived alone were allowed to form a “bubble” with another household to ease the negative effects of isolation (Department of Health and Social Care, U.G., 2020).*

### Procedure

The study was approved by the Science, Technology, Engineering, and Mathematics (STEM) Ethical Review Committee for the University of Birmingham (Ethics Approval Number: ERN_19-1176). Data was collected between February and April 2021, at a time where the United Kingdom was in its third national lockdown, with restrictions beginning to ease from March (Cabinet Office, 2021). All participants accessed the online questionnaire (hosted on SmartSurvey) and gave electronic informed consent before completing the study, following ethical guidelines outlined by the British Psychological Society.

### Materials

The following measures were included in the study:

#### Questionnaire for Assessing the Impact of the COVID-19 Pandemic and Accompanying Mitigation Efforts on Older Adults

The questionnaire for assessing the impact of the COVID-19 pandemic and accompanying mitigation efforts on older adults (QAICPOA, Cawthon et al., 2020) is a newly developed questionnaire that aims to assess the impact of the COVID-19 pandemic on the lives of older adults. It is formed of 17 questions, including the UCLA 3-item loneliness scale (Hughes et al., 2004) which is described in more detail below. The QAICPOA includes items on COVID-19 diagnosis and symptoms, actions taken in response to the pandemic (e.g., social distancing measures) and changes to frequency of communication with others. In addition to this questionnaire, we asked participants if they had received a COVID-19 vaccine, and if so, how many and when their last vaccination was.

#### Cognitive Health Worries

To establish levels of cognitive health worry, participants were asked the question “Are you worried about your cognitive health (abilities such as remembering things, communicating, doing and planning simple tasks)?” This question used “Yes, a lot,” “Yes, a little,” “Not really worried” and “Not at all worried” as potential answers.

#### Multidimensional Fatigue Inventory

The 20-item Multidimensional Fatigue Inventory (MFI-20, Smets et al., 1995) was used to assess five different aspects of fatigue: general fatigue, mental fatigue, physical fatigue, reduced activity, and reduced motivation. Items such as “I feel fit” and “I tire easily” are rated on a scale from 1–“Yes, that is true” to 5 – “No that is not true.” Scores can range from 5–20 per aspect of fatigue (possible total value of 100) with a higher score indicating higher levels of fatigue. Scores were calculated for each subscale, recoding negatively formulated items, giving five scores per participant. The scale has been shown to be a valid measure of fatigue (Smets et al., 1995).

#### Subjective Vitality Scale

The six-item version of the Subjective Vitality Scale (SVS, Ryan and Frederick, 1997) was used to measure levels of vitality. Respondents are asked to rate how true they believe statements to be in their general lives. Each item (for example “I have been feeling alive and vital” and “I nearly always feel alert and awake”) is rated on a six-point scale (from 1 – “Not at all true” to 6 – “Very true”). Scores are added up (out of 36) and calculated as an average of the six questions, with the highest score being 6. The scale demonstrates good levels of reliability in reported populations (Castillo et al., 2017). Higher scores indicate higher subjective vitality.

#### Patient Health Questionnaire-9

The Patient Health Questionnaire-9 (PHQ-9, Kroenke et al., 2009) is a nine-item questionnaire assessing symptoms of depression. Participants are asked “Over the past 2 weeks, how often have you been bothered by any of the following problems?” Participants then score statements, such as “Feeling down, depressed, or hopeless” or “Poor appetite or overeating” on a 4-point scale ranging from 0 – “Not at all,” to 3 – “Nearly every day,” resulting in a possible score of 27. Higher scores indicate more depressive symptoms. Scores of 5, 10, 15, and 20 indicate mild, moderate, moderate-severe, and severe depressive symptoms, respectively (Urtasun et al., 2019). It has been validated in clinical settings (Gilbody et al., 2007) and the general population (Martin et al., 2006).

#### Generalised Anxiety Disorder-7

The Generalised Anxiety Disorder-7 (GAD-7, Spitzer et al., 2006) is a seven-item questionnaire on anxiety symptoms. It asks participants to rate how often in the past 2 weeks they have been bothered by the following problems. Items such as “Not being able to stop or control worrying” or “Becoming easily annoyed or irritable” are scored on a 4-point scale from 0 – “Not at all,” to 3 – “Nearly every day,” giving a possible score of 21. Higher scores indicate higher levels of anxiety, with cut off points of 5, 10, and 15 indicating mild, moderate, and severe anxiety, respectively (Spitzer et al., 2006). The scale shows high validity and reliability when used with the general population (Lowe et al., 2008).

#### UCLA 3-Item Loneliness Scale

The UCLA 3-item Loneliness Scale (UCLA-3, Hughes et al., 2004) was included to measure levels of loneliness. The three included questions, “How often do you feel that you lack companionship?” “How often do you feel left out?” and “How often do you feel isolated from others?” were taken from the original, non-abbreviated scale (Russell, 1996) and have good internal consistency (Hughes et al., 2004). The questions are scored on a 3-point scale ranging from 1 – “Hardly ever” to 3 – “Often,” giving a maximum score of nine. A score of six or above has been used to classify participants as “lonely” (Steptoe et al., 2013).

#### Lubben Social Network Scale

The Lubben Social Network Scale-6 (LSNS-6, Lubben et al., 2006) is a 6-item questionnaire that measures social isolation in older adults by quantifying the frequency of social contact the respondent has from both friends and family, and the perceived support they get from these interactions. Questions include “How many relatives do you see or hear from at least once a month?” and “How many friends do you feel at ease with that you can talk about private matters?”. Each item is scored from 0 – “None,” to 5 – “Nine or more,” with a score of 12 or fewer indicating respondents being possibly at-risk of social isolation. The scale has been validated in older populations (Lubben et al., 2006).

#### Quality of Life and Sleep

Participants were asked “How would you rate your quality of life?” (rated on a 5-point Likert from 1 – “Very poor” to 5 – “Very good”), taken from the World Health Organisation Quality of Life Questionnaire (WHO, 2012), and “How would you rate your quality of sleep overall?” (rated on a 5-point Likert from 1 – “Very poor” to 5 – “Very good”) taken from the Pittsburgh Sleep Quality Index (Buysse et al., 1989).

#### The International Physical Activity Questionnaire Modified for the Elderly

The International Physical Activity Questionnaire for the Elderly (IPAQ-E) is a modified short version of the International Physical Activity Questionnaire (Craig et al., 2003). Participants are asked how often in the last week they spent sitting, walking, doing moderate physical activity and doing vigorous physical activity. Answers were given in days, and more specifically hours and minutes. Scores were converted into a continuous score of total MET (metabolic equivalent) per week as per scoring guidelines (Forde, 2018). Both the original IPAQ and the IPAQ-E have shown good levels of validity in reported populations (Hurtig-Wennlöf et al., 2010; Rubio Castañeda and Aznar, 2017).

### Data Reduction and Analysis

In addition to the measures above, we asked participants about their sitting activities, pain levels, sleep satisfaction and duration, health satisfaction, and information on social interaction and language use while doing physical activity. However, these measures were not included in the present analysis. Further questions about perceived cognitive health, including data on current and previous use of cognitive training methods, were also excluded.

Data were analysed using SPSS v26. Independent one-way ANOVAs were conducted to examine differences in wellbeing measures across levels of cognitive health worry. Next, Pearson correlations were conducted to look at relationships between all continuous variables (measures of depression, anxiety, loneliness, social isolation, age, fatigue, subjective vitality, and physical activity). Kruskal–Wallis tests were completed to examine differences in loneliness scores across living arrangements, and to examine the differences between COVID-19 concern and wellbeing measures.

## Results

### Cognitive Health Worries

In total, 2 (1%) participants were worried a lot about their cognitive health, 46 (24.1%) were a little worried, 73 (38.2%) were not really worried and 70 (36.6%) were not at all worried. For analysis and in all future reference to this question, “Yes, a lot” and “Yes, a little” were recoded into one group (“Yes, worried”).

A series of independent one-way ANOVAs using cognitive health worries as the independent variable (groups being “Yes, worried,” “Not really worried” and “Not at all worried”) were conducted to investigate differences in wellbeing. Significant group differences were found in scores for depression, anxiety, general fatigue, subjective vitality, loneliness, sleep quality, and quality of life. Physical fatigue, reduced activity, mental fatigue and reduced motivation (subsets of the MFI-20) are not included in this reported analysis, but all had similar significant group differences. Results, including individual group differences, are summarised in Table 2. Overall, participants who were worried about their cognitive health had poorer psychological wellbeing, were more fatigued, and less satisfied with sleep, health, and quality of life. Tukey *post hoc* analyses were completed for all significant results (see Table 2).

**TABLE 2:** One way ANOVA results.

Outcome measure	Cognitive health worries			*F*-value	*P*-value
	Yes, worried (*N* = 48)	Not really worried (*N* = 73)	Not at all worried (*N* = 70)		
Depression (PHQ-9)	5.87 (4.79)	3.45 (4.10)_a_	1.58 (2.39)_a, b_	18.13	<0.001
Anxiety (GAD-7)	4.29 (4.36)	2.42 (3.29)_a_	0.99 (2.19)_a, b_	14.36	<0.001
General fatigue (MFI-20)	12.26 (4.15)	10.24 (4.27)_a_	7.83 (3.63)_a, b_	17.68	<0.001
Subjective vitality (SVS)	3.18 (1.24)	3.75 (1.21)_a_	4.70 (1.26)_a, b_	23.26	<0.001
Loneliness (UCLA-3)	5.19 (1.89)	4.45 (1.63)_a_	4.03 (1.55)_a_	6.81	0.001
Sleep quality	3.13 (1.00)	3.33 (1.00)	3.72 (0.80)_a, b_	6.41	0.002
Quality of life	3.63 (0.73)	3.95 (0.83)	4.16 (0.83)_a_	6.18	0.003

*Data presented as mean (SD). DF = 184, 2. Statistical analyses were conducted using a one-way ANOVA. ^a^Significantly different from “Yes, worried” group. ^b^Significantly different from “Not really worried” group.*

### Psychological and Physical Wellbeing

The sample mean scores for the wellbeing questionnaires are reported in Table 3. Using clinical cut-off points mentioned above, 51 participants (26.8%) classified as lonely, and 41 (22.3%) participants were at risk of social isolation. Forty-four (23.5%) participants had mild depression, 8 (4.3%) moderate and 4 (2.1%) moderate-severe depression, while 27 (14.4%) scored mild anxiety, 10 (5.4%) moderate, and one (0.5%) severe anxiety.

**TABLE 3:** Descriptive statistics for variables.

Variable	Possible scores	Mean (SD)
Age	60–88	70.56 (6.41)
Sleep quality	1–5	3.42 (0.96)
MFI general fatigue	4–20	9.84 (4.36)
MFI physical fatigue	4–20	9.72 (4.61)
MFI reduced activity	4–20	9.30 (4.32)
MFI mental fatigue	4–20	7.83 (3.77)
MFI reduced motivation	4–20	7.67 (3.14)
SVS	1–7	3.96 (1.37)
PHQ-9	0–27	3.37 (4.10)
GAD-7	0–21	2.38 (3.50)
UCLA-3	0–9	4.48 (1.72)
LSNS-6	0–30	16.70 (4.98)
IPAQ-E total MET	–	2871.18 (2657.74)
Sleep quality	1–5	3.42 (0.96)
Quality of life	1–5	3.94 (0.83)

Pearson correlations were run on all continuous variables (see Table 4). A Bonferroni adjusted alpha level of 0.00125 was used. Overall, psychological wellbeing (depression, loneliness, and anxiety) was negatively correlated with physical activity, sleep quality and social network, and positively correlated with fatigue. More physical activity (in the IPAQ-E) was negatively correlated with all five facets of fatigue and depression (all *p* < 0.001), and positively correlated with subjective vitality. The size of social network was negatively correlated with loneliness and depression (*p* < 0.001), with bigger social networks being better for psychological health. Sleep quality was negatively correlated with fatigue, depression and anxiety, and positively correlated with subjective vitality (all *p* < 0.001).

**TABLE 4:** Correlation matrix and descriptive statistics for continuous variables.

	Age	Sleep quality	MFI general fatigue	SVS	PHQ-9	GAD-7	UCLA-3	LSNS-6	IPAQ-E total MET
Age	–								
Sleep quality	–0.051	–							
MFI general fatigue	0.146	−0.550*	–						
SVS	–0.136	0.499*	−0.623*	–					
PHQ-9	–0.061	−0.606*	0.667*	−0.645*	–				
GAD-7	–0.101	−0.389*	0.455*	−0.491*	0.722*	–			
UCLA-3	–0.086	–0.162	0.297*	−0.432*	0.421*	0.357*	–		
LSNS-6	–0.033	0.151	–0.143	0.193	−0.258*	–0.145	−0.299*	–	
IPAQ-E total MET	–0.044	0.215	−0.310*	0.337*	−0.256*	–0.048	–0.011	0.067	–

*p < 0.001. MFI Physical Fatigue, Reduced Activity, Mental Fatigue and Reduced Motivation subscales are not included in this table, but all showed similar significant relationships.

To further investigate the associations between loneliness and social isolation and wellbeing, participants were recoded into three groups of living arrangements: living alone (*N* = 25), living alone but in a support bubble (*N* = 11) and living with others, including family, spouses, etc., (*N* = 154). Because participant numbers were uneven across groups, a Kruskal-Wallis test was performed. Using living arrangements as the independent variable, the test revealed a significant difference between groups in scores of loneliness, χ^2^(2) = 16.46, *p* = < 0.001. *Post hoc* Dunn comparisons (using a Bonferroni adjusted *p*-value of 0.017) revealed that scores on the UCLA-3 item loneliness scale were significantly lower for participants who lived with other people (Mdn = 4) compared to those who lived alone (Mdn = 5, *p* = 0.007) and those who lived alone but were in a support bubble (Mdn = 5, *p* = 0.009). No significant difference was found between participants who lived alone and those who were part of a support bubble (*p* = 0.464). No other significant differences in psychological wellbeing scores were found (all *p* > 0.05).

### COVID-19 Concern

In answer to the question “How concerned are you about the COVID-19 pandemic?” Taken from the QAICPOA (Cawthon et al., 2020), five participants were “not at all concerned,” 81 were “somewhat concerned” and 104 “very concerned.” Due to uneven group sizes, a non-parametric Kruskal–Wallis test was completed to assess differences in groups. Using COVID-19 concern as the independent variable, results revealed a significant difference for General Fatigue (MFI-20) scores, χ^2^(2) = 6.41, *p* = 0.041, but after adjusting for multiple comparisons it was no longer significant. No significant differences were found in any other psychological wellbeing measures (PHQ-9, GAD-7, LSNS-6, UCLA-3, SVS, MFI-20; all *p*-values >0.05).

## Discussion

The present study investigated factors that are associated with wellbeing in the context of the COVID-19 pandemic in a sample of older adults in the United Kingdom. The main aim was to establish whether increased concern about cognitive abilities was related to poorer psychological and physical wellbeing. We further aimed to corroborate previous research undertaken during the pandemic that showed that higher levels of social contact and physical activity are associated with better psychological and physical wellbeing. Our final aim was to establish whether COVID-19 concern was associated with poorer psychological wellbeing. Our results showed that cognitive worries were significantly related to poorer psychological and physical wellbeing, while higher levels of physical activity and social contact were related to better wellbeing scores. Results also showed that psychological wellbeing scores did not differ between those that were very concerned about the COVID-19 pandemic and those that were not or somewhat concerned.

### Cognitive Health Worries

Out of our sample, 25.1% were at least somewhat concerned about their cognitive health. This is consistent with previous studies that have reported dementia worry at 26–60% of people in the population (Kessler et al., 2012; Bowen et al., 2019). Previous research has associated dementia worry with poorer wellbeing in the form of depression, stress, and life satisfaction (Cutler and Bragaru, 2017). Our results demonstrate that not only do cognitive health worries relate to psychological health in the form of depression, anxiety, and loneliness scores, but this extends to physical wellbeing. Participants who worried about their cognitive health were significantly more fatigued and reported poorer sleep quality and poorer self-reported quality of life.

We have shown a clear distinction between those who are worried about their cognitive health and those that are not, in terms of both psychological and physical wellbeing. Assuming there is a causal relationship between cognitive health worries and psychological wellbeing, finding ways of reducing these cognitive health worries will benefit wellbeing in this population. For example, a modified cognitive behavioural therapy (CBT) programme has been used successfully to reduce dementia worry in a sample of older adults (An et al., 2020), but it did not include measures of wellbeing. A potential alternative could be a cognitive training programme; hypothetically, improving cognitive functioning could lessen cognitive health worries, if the participant believes that the cognitive training is working. While the present study was not able to assess this due to the nature of the online study, a future cognitive training study plans to investigate this further. By exploring how cognitive worries change after a cognitive training programme and looking into how these relate to psychological and physical wellbeing, we may be able to determine a causal link between cognitive training, cognitive health worries, and wellbeing. We may also be able to determine whether cognitive worries are directly related to cognitive performance.

### Physical Activity and Social Interaction

Results show that participants who completed more physical activity reported significantly lower depression and fatigue scores. Similarly, participants with bigger social networks and had more regular contact with friends and family scored lower on depression and loneliness scales. This is in line with previous research conducted during the pandemic showing positive associations of physical activity (Carriedo et al., 2020) and social contact (Groarke et al., 2020) on mental health. Encouraging physical activity and social contact is therefore beneficial to promote during periods of social isolation.

Clinical cut-offs would suggest that 29.9% of our population were mild to moderately-severely depressed, 20.3% were mild to severely anxious, and that 26.8% classified as lonely (Spitzer et al., 2006; Steptoe et al., 2013; Urtasun et al., 2019). This is consistent with rates of mental health disorders in previous research conducted during the pandemic (Groarke et al., 2020; Rajkumar, 2020). Loneliness scores did not significantly differ between participants who lived alone and those that lived alone but were in a support bubble. Furthermore, both groups had higher loneliness scores than those who lived with other people. This supports research conducted during the pandemic that showed people who co-habit report lower levels of loneliness (Groarke et al., 2020). It suggests that while support bubbles may be valuable for some aspects of psychological wellbeing, they do not necessarily help to reduce feelings of loneliness. Lockdown loneliness has become prevalent during the pandemic (Shah et al., 2020) and so methods are needed to help combat this. Our results show that physical activity and social contact are related to better wellbeing scores, so guidelines and programmes that encourage physical activity with other people would be helpful to combat the negative effects of social isolation.

### COVID-19 Concern

Concern about the COVID-19 pandemic was not related to any significant differences in scores of psychological wellbeing scores; participants who were very concerned about the pandemic did not score higher on scores on anxiety or depression, as has been found previously (Aslam et al., 2021). This could be due to several contextual factors. Recruitment took place during easing of the United Kingdom lockdown (February to April 2021). While speculative, it may be that our sample felt positive as cases and deaths in the United Kingdom were declining at the time of recruitment (Riley et al., 2021), and only one participant reported to have been diagnosed with the virus. Importantly, all participants had received at least one vaccine at the time of recruitment; recent research has shown that participants who have had a COVID-19 vaccine show lower anxiety and depression symptoms compared to before being vaccinated (Perez-Arce et al., 2021). As all our participants had been vaccinated, we were not able to compare psychological wellbeing symptoms to unvaccinated participants to confirm this hypothesis.

### Limitations

The sample was primarily (95.8%) White. Black, Asian, and minority ethnic (BAME) groups are disproportionately affected by COVID-19 and see higher rates of infection and death (Iacobucci, 2020). Some disparities in mental health symptoms have already been found when comparing BAME to White British individuals during the pandemic, particularly for men (Proto and Quintana-Domeque, 2021). Future research could focus on older adult BAME communities to see whether these disparities remain.

Second, the sample size was relatively low compared to some large-scale studies investigating wellbeing in the pandemic (e.g., Robb et al., 2020; Shevlin et al., 2020), and we did not achieve the full number and representation of participants that we were originally aiming for. Approximately 20% of participants (51) were removed from our analysis due to incomplete survey responses, in an effort to reduce the risk of bias on results. Furthermore, our sample included a low number of oldest-old (80 + years) participants compared to youngest-old. Research has shown that in a sample of adults over 50, the risk of reporting worsening depression and anxiety symptoms as a result of the pandemic decreases with age, in that youngest-old participants report poorer wellbeing compared to oldest-old (Robb et al., 2020). Future research would benefit by examining age differences in psychological wellbeing in older adults during the COVID-19 pandemic, in a large, representative sample.

Finally, the study is cross-sectional, meaning that inferences cannot be compared over different time points. Recruitment took place almost 12 months into the pandemic, by which time the United Kingdom had gone through three national lockdowns. However, when recruitment started, the United Kingdom Governments “roadmap” out of lockdown had been published (Cabinet Office, 2021), restrictions were beginning to ease, and as mentioned above, cases were declining (Riley et al., 2021). This period is associated with a general improvement in psychological wellbeing scores in the United Kingdom general population, with scores fluctuating over time, coinciding with the number of restrictions in place and the number of cases in the United Kingdom (Daly and Robinson, 2021). Comparing findings with data collected when restrictions were toughest would be particularly interesting, to see whether cognitive health worries follow the same pattern.

## Conclusion

Results from the present study showed that cognitive health worries are associated with poorer psychological and physical wellbeing, across a range of measures. We also found that higher levels of physical activity and social contact are related to better psychological wellbeing, supporting previous research conducted during the pandemic. Results appeared to show no association between COVID-19 concern and any wellbeing measures, though this may be explained by vaccine status.

This research shows we need to continue to consider the role of regular physical activity and social contact on wellbeing, especially in those populations that may feel uneasy about re-entering society. It also highlights we need to focus resources on those who are worried about their cognitive health. By encouraging activities that can enhance cognitive functioning, perhaps we can lessen this concern.

## Data Availability Statement

The raw data supporting the conclusions of this article will be made available by the authors, without undue reservation.

## Ethics Statement

The studies involving human participants were reviewed and approved by the Science, Technology, Engineering, and Mathematics (STEM) Ethical Review Committee for the University of Birmingham. The patients/participants provided their written informed consent to participate in this study.

## Author Contributions

ES, JC, KS, and JVvZ: conception and design of the study. ES: data acquisition. ES, JC, and JVvZ: data analysis. All authors contributed to the data interpretations and drafting and final approval of the manuscript.

## Conflict of Interest

The authors declare that the research was conducted in the absence of any commercial or financial relationships that could be construed as a potential conflict of interest.

## Publisher’s Note

All claims expressed in this article are solely those of the authors and do not necessarily represent those of their affiliated organizations, or those of the publisher, the editors and the reviewers. Any product that may be evaluated in this article, or claim that may be made by its manufacturer, is not guaranteed or endorsed by the publisher.
